# Enhancing Student Wellbeing Through Social Prescribing: A Rapid Realist Review

**DOI:** 10.3389/phrs.2023.1605189

**Published:** 2023-08-08

**Authors:** Mark Davies, Megan Elliott, Sarah Wallace, Carolyn Wallace

**Affiliations:** ^1^ PRIME Centre Wales, University of South Wales, Pontypridd, United Kingdom; ^2^ Wales School for Social Prescribing Research (WSSPR), University of South Wales, Pontypridd, United Kingdom; ^3^ Welsh Institute for Health and Social Care, University of South Wales, Pontypridd, United Kingdom

**Keywords:** social prescribing, realist review, higher education, student support, wellbeing

## Abstract

**Objectives:** A Rapid Realist Review of social prescribing in Higher Education (HE) was undertaken to determine what works, for whom, how, why, and within what circumstances. The review resulted in the development of a Realist Programme Theory articulating the way in which social prescribing can be implemented within the HE environment.

**Methods:** Searches of 12 electronic databases were supplemented by citation chaining and grey literature surfaced by the Project Advisory Group. The RAMESES Quality Standards for Realist Review were followed, and the retrieved articles were systematically screened and iteratively analysed to develop Context-Mechanism-Outcome Configurations (CMOCs) and an overarching Realist Programme Theory.

**Results:** A total of 57 documents were included. The overarching programme theory was developed from the analysis of these documents and comprised of a social prescribing pathway with the following components: (1) An Accessible Gateway, (2) A Skilled Peer, (3) Trusted-Safe-Credible Resources, and (4) A Healthy Setting.

**Conclusion:** A Realist Programme Theory was developed—this model and associated principles will provide a theoretical basis for the implementation of social prescribing pathways within higher education. Whilst the direct project outputs are of particular significance to the UK HE audience, the underpinning principles can support practice within the global arena.

## Introduction

Within the United Kingdom (UK), it is estimated that up to 20% of Primary Care appointments are for social or economic, rather than medical reasons [1–3]. Mental health issues are now more prevalent within the general population, a situation that has been exacerbated by the COVID-19 pandemic [4, 5]. UK policy and legislative documents have called for new and innovative services to address these challenges [6–8], indicating that resources should deliver holistic support for citizens which promote health and wellbeing.

Social prescribing is a globally evolving initiative [9] which has the potential to address these pressures and prevent future ill health. Models of social prescribing are heterogeneous and highly complex [10, 11], varying in structure, intensity of service provision, and complexity [12]. Models typically comprise a link worker, also known as a community connector, navigator, or wellbeing advisor, who works with an individual to identify goals and needs and connect them with non-medical community resources [13, 14]. A range of models have emerged over the past decade, varying in structure, complexity, and intensity of provision [15]. Services include informal signposting to community resources and assets, through to holistic support where link workers work closely and co-productively with the individual over a series of appointments to identify resources to meet their needs [12].

Social prescribing interventions have been targeted at a range of population groups and conditions including people who are socially isolated, older individuals experiencing sensory impairment, and those with long term health issues [14, 16–20], but Higher Education (HE) students and young people remain a demographic where further investigation regarding the implementation of social prescribing is warranted. Student wellbeing levels are considerably lower than the general population [21], and marked increases in student wellbeing issues have been observed in recent years [22]. Factors including change of locality, the pressures of independent living and learning, new personal responsibilities all contribute to reduced wellbeing and challenges of adjustment are particularly amplified for mature students, those with declared disability, and learners from Black and Asian Minority Ethnic groups [21, 23–25].

In addition, the student population continue to be considerably affected by the COVID-19 pandemic, impacting on social isolation, stress, anxiety, loneliness, social interactions, and wellbeing [26–28]. Whilst several strategies have been developed [29], effectively supporting student wellbeing remains difficult. Given the struggles experienced by this specific group and the large and increasing number of individuals attending HE [25], the potential for social prescribing within this environment merits further exploration.

This Rapid Realist Review seeks to evaluate social prescribing in HE—informing the development of a service model which will effectively meet the varying needs of a range of individuals. To achieve this aim, five specific review questions were devised:

1. *What* forms of social prescribing interventions are specifically targeted at HE students?
2. *How* do HE students access social prescribing interventions targeting them?
3. *When* do HE students access social prescribing interventions?
4. *For whom* do social prescribing interventions work?
5. *To what extent* does social prescribing work for HE students?

## Methods

Conventional approaches to the evaluation of interventions such as the systematic review focus predominantly upon outcome, i.e., *what works* [30]. In order to understand how a scalable social prescribing pathway may be developed for students in HE, it is imperative to appreciate the impact of context; to understand *how*, *why*, *for whom*, *to what extent*, and *in what context* the intervention works [31]. Realist methodology satisfies these demands; it is a theory-driven approach to the synthesis of evidence, the goal of which is to build a programme theory that explicates what the intervention is, and how it can be expected to work. The Realist Review method is grounded within generative causation, meaning that to infer a causal relationship between an intervention (I) and outcome (O), one must understand the underpinning mechanism (M) connecting them, as well as the context (C) in which they occur. Using the Realist approach, we have unpacked the Context-Mechanism-Outcome configurations (CMOCs) underpinning existing pathways, and in doing so, have built a Realist programme theory of social prescribing for students in HE.

### Developing the Project Scope

Refining the focus of the review and developing scope took place collaboratively with an Advisory Group [32] comprising key student services personnel, student representatives, content experts, 3rd Sector partners, and members of the research team from across both institutions. A Population, Intervention, Comparison, Outcome (PICO), and search strategy were developed (Table 1), and informal scoping of existing evidence and policy was undertaken to pilot test search terms and build familiarity with existing knowledge relating to social prescribing. Whilst these initial searches indicated that literature focusing specifically upon the use of social prescribing with HE students was limited, examining similar interventions in which social prescribing had been employed, allowed the detection of demi-regularities [33, 34] or causal patterns that formed the basis of CMOCs within this project.

**TABLE 1:** PICO & Literature Searches (University of South Wales, United Kingdom. 2022).

PICO framework	
Patient/Population Under Study	HE Students
Intervention	Social Prescribing
Comparison	Students sourcing own wellbeing interventions
Outcome	Impact of specified Social Prescribing model upon overall student wellbeing
Search Terms	
Search Term	Alternate
Wellbeing	Wellbeing, Wellbeing, Wellness,
Resilience	Resilien*, Hardiness, Reserve, Coping,
Isolation	Isolat*, Separat*, Lonel*, Remot*, Living Away, Social Isolation
Relationships	Relationship*, Connection*,
Local Community	Locality, Neighbourhood, Community Group,
Lifestyle	Physical Health, Mental Health, Healthy Living,
Social Prescribing	Social Prescr*, Link Worker*, Link Navigator, Link Coordinator, Community Refer*, Community Connect*, Community Coordinator, Community Navigator, Community Champion, First Contact Practitioner, Local Area Coordinator, Social Capital, Community Asset*,
Student	Student*, Learner*, Part-time Student*, Rural Student*, Liberation Groups, Mature Student*, Students with Declared Disabil*,
Higher Education	HE, FE in HE, University, Tertiary Education
Self-Efficacy	Self-Management
Literature Sources	
Documents	
Primary Care Hub (2018) Social Prescribing in Wales: Final Report. Cardiff: Public Health Wales	
Social Services and Wellbeing (Wales) Act 2014	
Wellbeing of Future Generations (Wales) Act, 2015	
Welsh Government (2018) A Healthier Wales: Our Plan for Health and Social Care. Cardiff: Welsh Government	
Databases	
ASSIA, British Education Index, CINAHL, ERIC, Medline, ProQuest Psychology Journals, PsychInfo, PubMed, Science Direct, Scopus, Social Care Online, Web of Science	
Grey Literature	
Local Authority Websites, Third Sector Websites, University Websites, HE Sector Policy Documents, ‘OpenGrey’	
Inclusion & Exclusion Criteria	
Inclusion	Exclusion
English language	Students in FE or Continuing Education
Published literature	
Grey literature	
Students in HE	
Date Range	
All available entries up to April 2020	
Search Strings	
Concept 1 Wellbeing	
Wellbeing OR “Wellbeing” OR “Wellbeing” OR Wellness	
Concept 2 Resilience	
Resilien* OR Hardiness OR Reserve OR Coping	
Concept 3 Isolation	
Isolat* OR Separat* OR Lonel* OR Remot* OR “Living Away” Or “Social Isolation”	
Concept 4 Relationships	
Relationship* OR Connection*	
Concept 5 Local Community	
“Local Community” OR Locality OR Neighbourhood OR “Community Group”	
Concept 6 Lifestyle	
Lifestyle OR “Physical Health” OR “Mental Health” OR “Healthy Living”	
Concept 7 Social Prescribing	
“Social Prescr*” OR “Link Worker*” OR “Link Navigator” OR “Link Coordinator” OR “Link Co-ordinator” OR “Community Refer*” OR “Community Connect*” OR “Community Coordinator” OR “Community Co-ordinator” OR “Community Navigator” OR “Community Champion*” OR “First Contact Practitioner” OR “Local Area Coordinator” OR “Local Area Co-ordinator” OR “Social Capital” OR “Community Asset*”	
Concept 8 Student	
Student* OR Learner* OR “Part-Time Student*” OR “part time student*” OR “Rural Student*” Or “Liberation Groups” OR “Mature Student*” OR “Students with Declared Disabil*”	
Concept 9 Higher Education	
“Higher Education” OR HE OR “FE in HE” OR “Tertiary Education” OR University NOT FE	
Concept 10 Self Efficacy	
“Self Efficacy” OR “Self-Efficacy” OR “Self Management” OR “Self-Management”	
Search Outcomes	
Total Hits	4,338
Duplicates Removed	2,347
Including Grey Literature from Advisory Group	2,358
For Abstract Screen	349

### Search Strategy

**FIGURE 1:**
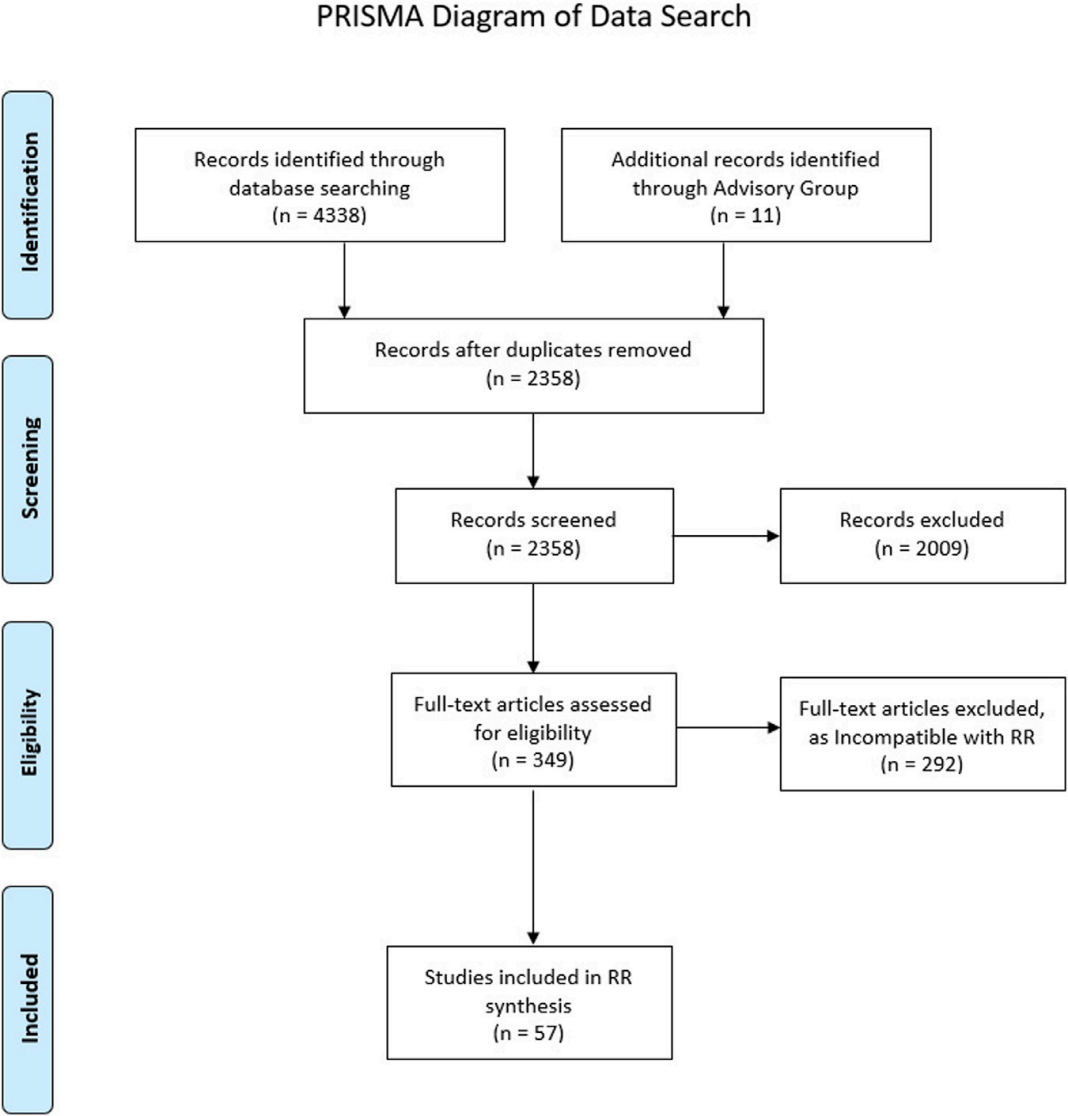
PRISMA diagram (University of South Wales, United Kingdon. 2021).

An Initial Programme Theory (IPT) should be the starting point within any Realist Review—encapsulating views of the research team and Advisory Group regarding how and why an intervention should work [35]. Search terms encompassing the IPT were initially drafted in conjunction with the Advisory Group, executed, and refined iteratively as the IPT was developed. Following further consultation with an information specialist, twelve databases were searched (Table 1). Grey literature obtained from Local Authority and 3rd Sector websites from the UK were additionally interrogated, and further documents were provided by the Advisory Group. Citation chaining was also used to identify other relevant documents throughout the review; however given that a Rapid Realist Review was being undertaken [36], there were limited opportunities to add documents to the review in this way. The search results were exported and de-duplicated within EndNote X9 (Clarivate Analytics, Philadelphia PA, USA) before being fully extracted into Microsoft Excel (Microsoft Corporation, Redmond WA, USA) for the research team to have full access. A PRISMA diagram outlining the search is presented within Figure 1.

### Selecting Documents

Documents were selected based on the degree to which they addressed the research questions, and ultimately contributed to the development of a Refined Programme Theory. Inclusion decisions were made by the full review team based upon *relevance* - how these data might contribute to theory building, and *rigour*—the degree to which the methods used to generate these data are plausible and trustworthy [34]. Titles retrieved from the searches were initially screened by MD for salience; any deemed irrelevant were excluded at this stage. An abstract screening tool was developed by the project team and utilised to screen the remaining documents and determine if they met the inclusion criteria (Table 1). Where it was unclear if the abstracts met the inclusion criteria, the full text document was retained for screening. Abstract screening was undertaken by MD and CW; ME ensured inter-rater reliability by screening 10% of the overall abstracts. Full text screening was completed by MD and CW to establish the final data set for inclusion, and this was transferred to NVivo 12 (QSR International, Warrington, UK) for coding. Again, 10% of the documents were screened by ME, and any disagreements were resolved through engagement and discussion with the project Advisory Group.

Existing evidence was interrogated to further confirm, refine or refute the IPT, leading to the development of an abstracted model explaining how and why our programme works, for whom, to what extent, and in what circumstances [34]. Through an iterative process of data collection, analysis and synthesis [32], CMOCs were built that continued to shape the emerging programme theory. Whilst qualitative appraisal frameworks such as the *CASP Qualitative Studies Checklist* [37] may have significant utility within conventional systematic reviews, they must be applied cautiously in a Realist Review in order to avoid excluding documents which could significantly impact upon programme theory development [32] - even documents that lack methodologically robustness may still potentially contain “nuggets” that could advance the theory building process [38]. Discussing the content of any potentially problematic document within the research team and Advisory Group was helpful in this respect, as was following the *RAMESES Quality Standards for Realist* Synthesis [39] and selecting literature that provided ‘conceptually rich’ accounts of phenomena [40].

### Extracting and Organising the Data

Once the data set was finalised (Table 2), full text documents were sourced and located within a shared drive accessible by all team members and transferred to NVivo 12 (QSR International, Warrington, UK) for coding. A data extraction tool using Microsoft MS Excel (Microsoft Corporation, Redmond WA, USA) was also employed to collate information regarding the authors, date, source, nature of intervention, potential CMOCs, overall themes and any reference to substantive theory.

**TABLE 2:** Final Data Set (University of South Wales, United Kingdom. 2022).

Authors	Date	Article title	Country	Study design	Sample/Setting	Objectives
Abbas, M. A. Eliyana, P. A. Ekowati, D. D. Saud, M. M. Raza, M. A. Wardani, M. R.	2020	Data set on coping strategies in the digital age: The role of psychological wellbeing and social capital among university students in Java Timor, Surabaya, Indonesia	Indonesia	Survey questionnaire	University students	To examine the effects of technology on coping strategies & psychological wellbeing
Agadullina, E. R. Lovakov, A. Kiselnikova, N. V.	2020	Does quitting social networks change feelings of loneliness among freshmen? An experimental study	Russia	Experimental design	Freshman psychology students	To examine the impact of quitting social networks upon loneliness and isolation
Alizadeh, Z. Safaian, A. Mahmoodi, H. Shaghaghi, A.	2018	Pathways between authentic happiness and health promoting lifestyle profiles of the university students in Tabriz, Iran	Iran	Cross-sectional study	Iranian university students	To explore the relationship between and other health promoting behaviours
Altinyelken, H. K.	2018	Promoting the psychosocial wellbeing of international students through mindfulness: A focus on regulating difficult emotions	Netherlands	Qualitative study	International students at a Dutch university	To explore the potential of a mindfulness programme for providing psycho-social support to international students in higher education
Anderson, S. Fast, J. Keating, N. Eales, J. Chivers, S. Barnet, D.	2017	Translating knowledge: Promoting health through intergenerational community arts programming	Canada	Community participatory research project	Older adults and university students	To explore the health and wellbeing benefits of participation in an intergenerational community arts programme
Ashbaugh, K. Koegel, R. Koegel, L.	2017	Increasing social integration for college students with autism spectrum disorder	United States	Experimental design	University students with a confirmed diagnosis of Autistic Spectrum Disorder	To assess whether a structured social planning intervention would increase social integration for college students with Autistic Spectrum Disorder
Bailey, K. M. Frost, K. M. Casagrande, K. Ingersoll, B.	2020	The relationship between social experience and subjective wellbeing in autistic college students: A mixed methods study	United States	Mixed methods study	University students who had identified themselves to the college’s disability resource centre	To explore the relationship between the college social experience and subjective wellbeing in autistic student in the Midwestern United States
Barsell, D. J. Everhart, R. S. Miadich, S. A. Trujilo, M. A.	2018	Examining health behaviours, health literacy, and self-efficacy in college students with chronic conditions	United States	Survey questionnaire	Undergraduate students from a Mid Atlantic US university with a self-identified chronic health conditions lasting >3 months	To examine associations between health literacy, self-efficacy, and health behaviours in a sample of college students with chronic conditions
Bertotti, M.Frostick, C. Hutt, P. Sohanpal, R. Carnes, D.	2018	A Realist Evaluation of social prescribing: an exploration into the context and mechanisms underpinning a pathway linking primary care with the voluntary sector	United Kingdom	Realist Evaluation	Users of social prescribing services	To use a Realist approach in order to evaluate a social prescribing pilot in the areas of Hackney and City in London.
Bird, A. Pincavage, A.	2016	A curriculum to foster resident resilience	United States	Case study	Medical students	To evaluate the impact of a ‘resilience curriculum’ upon resilience and wellness
Boda, Z. Elmer, T. Voros, A. Stadtfeld, C.	2020	Short-term and long-term effects of a social network intervention on friendships among university students	Switzerland	Field experiment & observational study	Undergraduate university students	To investigate the short-term and long-term effects of randomized first contact opportunities on friendship networks in an emerging community of first-year undergraduate students
Bouteyre, E. Maurel, M. Bernaud, J.	2006	Daily hassles and depressive symptoms among first year psychology students in France: The role of coping and social support	France	Survey questionnaire	First year undergraduate university students	To explore the impact of coping strategies upon daily hassles and depressive symptoms
Brunsting, N. C. Zachry, C. Liu, J. Bryant, R. Fang, X. Wu, S. Luo, Z.	2019	Sources of perceived social support, social-emotional experiences, and psychological wellbeing of international students	United States	Structural equation modelling	Graduate and undergraduate students	To test whether specific social influences could enhance international students’ belonging and wellbeing and attenuate loneliness
Budzynski-Seymour, E. Conway, R. Wade, M. Lucas, A. Jones, M. Mann, S. Steele, J.	2020	Physical activity, mental and personal wellbeing, social isolation, and perceptions of academic attainment and employability in university students: The Scottish and British active students’ surveys	United Kingdom	Cross sectional surveys	University and college students in Scotland and the United Kingdom	To explore the relationships between physical activity and personal wellbeing within UK university students
Byrom, N.	2018	An evaluation of a peer support intervention for student mental health	United Kingdom	Cohort study	Students within 8 UK universities	To identify students likely to attend peer support and evaluate the acceptability and impact of the intervention
Chandler, G. E. Kalmakis, K. A. Chiodo, L. Helling, J.	2020	The efficacy of a resilience intervention among diverse, at-risk, college athletes: a mixed-methods study	United States	Mixed methods study	US college athletes	To assess the efficacy of a strengths-based resilience intervention upon perceptions of stress, resilience, emotional awareness, and belonging among student-athletes.
Chow, K. M. Tang, W. K. Chan, W. H. Sit, W. H. Choi, K. C. Chan, S.	2018	Resilience and wellbeing of university nursing students in Hong Kong: A cross sectional study	China	Cross sectional descriptive correlation design	Chinese undergraduate nursing students	To explore resilience levels in nursing students and its relationship with wellbeing
Karishma, C. Armstrong, S. Bean, S.	2018	Supporting students facing mental health challenges	United States	Survey questionnaire	Graduate and undergraduate students	To assess anxiety levels in university students and consider potential support strategies
Daddow, A. Cronshaw, D. Daddow, N. Sandy, R.	2019	Hopeful cross-cultural encounters to support student wellbeing and graduate attributes in Higher Education	Australia	Qualitative programme evaluation	University students	To evaluate an extracurricular programme that enabled interfaith and cross-cultural dialogue
Denovan, A. Macaskill, A.	2017	Stress, resilience and leisure coping among university students: Applying the broaden-and-build theory	United Kingdom	Structural equation modelling	University students	To investigate whether resilience predicts leisure coping and positive affect and whether this relationship is predictive of higher levels of wellbeing
Donoghue, J. O’Rourke, M. Hammond, S. Stoyanov, S. O’Tuathaigh, C.	2020	Strategies for enhancing resilience in medical students: a Group Concept Mapping analysis	Ireland	Group Concept Mapping analysis	Undergraduate medical students	To use GCM software to explore and categorise resilience strategies employed by third year undergraduate medical students
Dunn, L. B. Moutier, C.	2008	A conceptual model of medical student wellbeing: Promoting resilience and preventing burnout	International	Literature Review	Undergraduate medical students	To review the literature on medical student stress, coping, and wellbeing in order to develop a model of medical student coping termed the “coping reservoir.”
Enrique, A. Mooney, O. Salamanca-Sanabria, A. Lee, C. T. Farrell, S. Richards, D.	2019	Assessing the efficacy and acceptability of an internet-delivered intervention for resilience among college students: A pilot randomised control trial protocol	Ireland	Randomised control trial (protocol)	University students	To evaluate a newly developed internet-delivered intervention for resilience provided with human or automated support
Galante, J. Dufour, G. Vainre, M. Wagner, A. P. Stochl, J. Croudace, T. J. Benton, A. Howarth, E. Jones, P. B.	2016	Provision of a mindfulness intervention to support university students’ wellbeing and resilience to stress: preliminary results of a randomised controlled trial	United Kingdom	Randomised control trial	University students	To assess the efficacy of an 8 week mindfulness course versus usual mental health provision
Gee, K. A. Hawes, V. Cox, N. A.	2019	Blue Notes: Using songwriting to improve student mental health and wellbeing. A pilot randomised controlled trial	United Kingdom	Wait-list randomised control trial	University students with self-identified anxiety	To assess the efficacy of a weekly songwriting programme versus no intervention
Gieck, D. J. Olsen, S.	2007	Holistic wellness as means to developing a lifestyle approach to health behaviour among college students	United States	Cohort study	University students	To examine the influence of a holistic model of wellness on activity level among obese and sedentary college students
Haas, J. Pamulapati, L. G. Koenig, R. A. Keel, V. Ogbonna, K. C. Caldas, L. M,	2020	A call to action: Pharmacy students as leaders in encouraging physical activity as a coping strategy to combat student stress	United States	Commentary paper	Undergraduate pharmacy students	This commentary is a call to action for student pharmacists to take shared ownership over improving the current crisis of student wellbeing - empower their students to guide the improvement of wellness.
Rand Health	2016	Campus Climate Matters: Changing the Mental Health Climate on College Campuses Improves Student Outcomes and Benefits Society.	United States	Research brief	University students	To discuss a CalMHSA prevention & early intervention programme targeting health and wellbeing
HEFCW	2019	HE for a Healthy Nation: Student Wellbeing and Health	United Kingdom (Wales)	Report	University students	Report examining a range of student wellbeing strategies.
Harrer, M. Adam, S. H. Baumeister, H. Cuijpers, P. Karyotaki, E. Auerbech, R. P. Kessler, R. C. Bruffaerts, R. Berking, M. Ebert, D. D.	2019	Internet interventions for mental health in university students: A systematic review and meta-analysis	International	Systematic review and meta-analysis	University students	To search for randomized trials examining psychological interventions for the mental health, well‐being, and functioning of university students
Herrero, R. Adriana, M. Giulia, C. Etchemendy, E. Banos, R. Garcia-Palacios, A. Ebert, D. D. Franke, M. Berger, T. Schaub, M. P. Goerlich, D. Jacobi, C. Botella, C.	2019	An Internet based intervention for improving resilience and coping strategies in university students: Study protocol for a randomized controlled trial	International	Randomised control trial (protocol)	University students	To evaluate the efficacy of an unguided internet-based intervention to enhance resilience in university students
Holt, M. Powell, S.	2017	Healthy Universities: a guiding framework for universities to examine the distinctive health needs of its own student population	United Kingdom	Survey questionnaire	University students	To examine the student health behaviours of one university so that future initiatives can be tailored to its own student population
Husk, K. Blockley, K. Lovell, R. Bethel, A. Lang, I. Byng, R. Garside R.	2019	What approaches to social prescribing work, for whom, and in what circumstances? A Realist Review	United Kingdom	Realist Review	Users of social prescribing services	To develop a programme theory articulating the ways in which social prescribing works, for whom, and in what circumstances
Hussain, R. Guppy, M. Robertson, S. Temple, E.	2013	Physical and mental health perspectives of first year undergraduate rural university students	Australia	Survey questionnaire	Rural university students	To examine the physical and mental health issues for first year Australian rural university students and their perception of access to available health and support services
Kampel, L. Orman, J. O’Dea, B.	2017	E-mental health for psychological distress in university students: A narrative synthesis on current evidence and practice	Australia	Narrative synthesis	University students	To outline the current knowledge and application of e-mental health programs, and to discuss ways that prevention and intervention programs delivered via the Internet and smartphones can be taken to scale to reach a larger number of students to improve their mental health
Knight, A. LaPlaca, V.	2013	Healthy Universities: taking the University of Greenwich Healthy Universities Initiative forward	United Kingdom	Commentary paper	University students	The paper sets out the background to the national Healthy Universities initiative, briefly outlines a pilot initiative; and ends by considering the broader developments in policy and practice
Lattie, E. G. Adkins, E. C. Winquist, N. Stiles-Shields, C. Wafford, E. Graham, A. K.	2019	Digital mental health interventions for depression, anxiety, and enhancement of psychological wellbeing among college students: systematic review	International	Systematic review	University students	To review the literature on digital mental health interventions focused on depression, anxiety, and enhancement of psychological wellbeing among samples of college students to identify the effectiveness, usability, acceptability, uptake, and adoption of such programs
Montagni, I. Cosin, T. Sagara, J. A. Bada-Alonzi, J. Horgan, A.	2020	Mental health-related digital use by university students: a systematic review	International	Systematic review	University students	To summarize and critique studies of mental health-related digital use by students worldwide, to support the implementation of future digital mental health interventions targeting university students.
Murr, A. H. Miller, C. Papadakis, M.	2002	Mentorship through advisory colleges	United States	Case study	Medical students	To outline the development and implementation of an advisory college system that supports medical student wellbeing
Oades, L. G. Robinson, P. Green, S. Spence, G. B.	2011	Towards a positive university	Australia	Commentary paper	University students	The paper explores the concept of the “Positive University.”
Owens, A. R. Loomes, S. L.	2010	Managing and resourcing a program of social integration initiatives for international university students: what are the benefits?	Australia	Mixed methods	International students studying at Australian universities	To report the results of a survey of 446 CQUniversity international students who have had access to enhanced social integration opportunities for integration as well as a focus-group discussion with staff and students
Palma-Gomez, A. Herrero, R. Banos, R. Garcia-Palacios, A. Castaneiras, C. Fernandez, G. L. Llull, D. M. Torres, L. C. Barranco, L. A. Cardenas-Gomez, L. Botella, C.	2020	Efficacy of a self-applied online program to promote resilience and coping skills in university students in four Spanish-speaking countries: study protocol for a randomized controlled trial	International	Randomised control trial (protocol)	University students	To evaluate the efficacy of an unguided internet-based intervention to enhance resilience in university students
Papadatou-Pastou, M. Campbelll-Thomas, L. Barley, E. Haddad, M. LaFarge, C. McKeown, E. Simeonov, L. Tzotzoli, P.	2019	Exploring the feasibility and acceptability of the contents, design, and functionalities of an online intervention promoting mental health, wellbeing, and study skills in Higher Education students	United Kingdom	Cohort study	Graduate and undergraduate students	To evaluate the effectiveness of this intervention protocol in comparison with an active control condition targeting healthy lifestyle, and a waiting list control condition
Primary Care Hub	2018	Social Prescribing in Wales: Final Report	United Kingdom (Wales)	Report	Users of social prescribing services	A report providing an overview of the evolution, efficacy, and implementation challenges of social prescribing in Wales
Randstad	2020	A Degree of Uncertainty: Student Wellbeing in Higher Education	United Kingdom	Survey	University students	To explore the perceptions of students regarding their perceived levels of support whilst studying in UK universities
Ray, E. C. Arpan, L. Oehme, K. Perko, A. Clark, J.	2019	Helping students cope with adversity: a test of the influence of a web-based intervention on students’ self-efficacy and intentions to use wellness-related resources	United States	Randomised control trial	Undergraduate university students	To assess the efficacy of an online student wellness intervention versus usual provision
Short, B. Lambeth, L. David, M. Ryall, M. A. Hood, C. Pahalawatta, U. Dawson, A.	2019	An immersive orientation programme to improve medical student integration and wellbeing	United States	Mixed methods	Medical students	To evaluate an immersive orientation programme aimed at promoting student wellbeing through social connectedness
Sibley, S. Sauers, L. Daltrey, R.	2019	Humanity and Resilience Project: The development of a new outreach program for counselling centres at colleges and universities	United States	Case study	University students	To present an overview of a programme developed to increase resilience by encouraging social connection within students at a US university
Stalman, H. M.	2019	Efficacy of the My Coping Plan mobile application in reducing distress: A randomised controlled trial	Australia	Randomised control trial	University students with self-reported elevated levels of distress	To assess the efficacy of an online strengths-focused coping plan app versus usual provision
Thomas, K. Bendsten, M.	2019	Mental Health Promotion Among University Students Using Text Messaging: Protocol for a Randomized Controlled Trial of a Mobile Phone-Based Intervention	Sweden	Randomised control trial (protocol)	University students	To test the efficacy of a mobile phone–based intervention on positive mental health
Thomas, S. S.	2009	Top 10 Strategies for Bolstering Students’ Mental Resilience	United States	Commentary paper	University students	Commentary paper outlining a series of strategies that may be effective in increasing student resilience levels
Thorley, C.	2017	Not by Degrees: Improving Student Mental Health in the UK’s Universities	United Kingdom	Report	University students	A report outlining setting forth the challenges surrounding student mental health and wellbeing, and outlining a series of interventions that may be helpful in addressing these
Tierney, S. Wong, G. Roberts, N. Boylan, A. Park, S. Abrams, R. Reeve, J. Williams, V. Mahtani, K. R.	2020	Supporting social prescribing in primary care by linking people to local assets: a Realist Review	United Kingdom	Realist Review	Users of social prescribing services	To understand how such social prescribing connector schemes work, for whom, why and in what circumstances
Wawera, A. S. McCamley, A.	2019	Loneliness among international students in the UK	United Kingdom	Mixed methods	International students at UK universities	To explore loneliness in an international student population in a single university
Webster, N. L. Oyebode, J. R. Jenkins, C. Bicknell, S. Smythe, A.	2020	Using technology to support the emotional and social wellbeing of nurses: A scoping review	United Kingdom	Scoping review	Undergraduate nursing students	To review the literature on the use of technology to offer emotional and social support to nurses
Welsh Government	2018	A Healthier Wales: Our Plan for Health and Social Care	United Kingdom (Wales)	Strategy Document	Population of Wales	The document sets out a long term future vision of a whole system approach to health and social are which is focussed upon health and wellbeing, and on preventing illness
Wiljer, D. Johnson, A. McDiarmid, E. Abi-Jaoude, A. Ferguson, G. Hollenberg, E. Van Heerwaarden, N. Tripp, T. Law, M.	2017	Thought Spot: Co-Creating Mental Health Solutions with Post-Secondary Students	Canada	Case study	University students	To explore the development, utilisation, and impact of a web-based platform that supports students in seeking mental health and wellbeing support

### Synthesizing the Evidence and Building the Programme Theory

Full text was first examined by MD who identified initial themes or tentative ‘bucket codes’ that may reflect a Context, Mechanism, Outcome, or any combination of these. This process continued iteratively as CMOCs were developed, allowing the emerging Programme Theory to be supported, refined, or refuted. CW simultaneously double coded 20% of the data set and both reviewers discussed and agreed the emerging CMOCs. The process was further supported by ME, who was involved in all discussions regarding coding, the development of CMOCs, and the Refined Programme Theory.

The initial bucket codes remained very broad but proved to be a useful stepping-stone as we transitioned from analysis to synthesis. Three key reasoning strategies were employed synergistically to facilitate data analysis, synthesis, and theory building. Careful “observation” of the data resulted in the *inductive* generation of codes, whilst themes or propositions from the initial programme theory/substantive theory were tested against the data in a *deductive* manner to build CMOCs. Finally, *retroductive* reasoning involved using both induction and deduction as well as drawing on one’s owns insights in a manner consonant with the abductive reasoning of Peirce [41] in order to elucidate causal mechanisms [42]. A full list of CMOCs can be found within Table 3.

**TABLE 3:** CMOC List (University of South Wales, United Kingdom. 2022).

Theme	CMOC No (Paper):	CMOC
Accessible Gateway	23	When interventions and/or services are perpetually available online (C), Students feel that there is always support (M), and this increases their sense of safety and overall wellbeing (O)
	49	When students have access to an app that enables them to view a range of alternative wellbeing provision (C), They will engage with healthy coping resources (M) and develop an individual coping plan (O)
	57	When a Social Prescribing pathway is linked to existing IT platforms (C), stakeholders will perceive it as easy to use (M) and engage more readily (O)
	38	When services provide HE students who are vulnerable with useful digital resources (C) they are more likely to engagement with these (O), because they do not feel judged or stigmatised by anyone (M)
	46	When web-based interventions link students into campus resources (C), the students feel less stigmatized (M), and there is greater uptake of Mental Health and Wellbeing services
	29	When resources are made available on-site simultaneously from the university and other partners (C), students feel a greater sense of agency (M) becoming aware of the support available (O)
	29	When student wellbeing champions are present at high traffic student ‘hotspots’ (C), there is a sharing of resources (M), an increased awareness of the help that is available (O)
Skilled Peer	51	When the student is confident that their needs will be met (M) by a *Gatekeeper’s* knowledge of early interventions (C), they will engage with the service (O)
	57	When navigators demonstrate a genuine desire to help by offering personalised support (C), users will believe that they can benefit from the interaction (M) and engage with the service and subsequent Social Prescription (O)
	52	When trusted friends, tutors and parents are involved when students are struggling with wellbeing (C), there is a normalising of stressful experiences (M) and a subsequent building of resilience and improved wellbeing (O)
	57	When establishing the Social Prescribing pathway (C), third sector and community services will buy into the navigator role (O), if they believe that the referrals received are appropriate (M)
	57	When a user perceives the navigator to be part of a formal mental health or social services structures (C), a fear of stigmatising (M) may prevent them engaging with the services (O)
	52	When peer support is provided by student volunteers (C), there is an externalising of MH and wellbeing issues (M), and students deal more effectively with mental health & wellbeing challenges (O)
	16	When student volunteers provide wellbeing peer support (C), they share coping strategies (M), building and maintains positive mood (O)
	27	When students engage in peer-led wellness activities (C), they feel a shared connection and view the activities to be authentic and trustworthy (M), and this increases engagement (O)
	57	Developing systems providing supervision or peer support (C), enables anxieties regarding the role to be shared and explored (M), buffering the psychological pressure the navigator may experience (O)
Trusted-Safe-Credible Resources	38	When credible digital resources are provided (C) students will have confidence and trust in them (M) with consequent high levels of uptake and sustained engagement (O)
	34	When a library of online self-help resources is provided (C), students will believe that self-improvement is possible (M), and their agency and self-efficacy will increase (O)
	56	When students digitally curate community-based wellbeing resources (C), they share their knowledge with peers (M), building support networks and increasing social capital
	07	When students and older community members come together and collaborate on a shared activity (C), social connections are built between the university and wider community (M), and there is increased social capital and associated wellbeing for all parties (O)
	58	When a referral is made to a community asset (C), the service user has opportunities to meet and share experience with those within and beyond the university (M), building their social capital
	57	When developing a Social Prescribing pathway (C), third sector and community services should be active co-producers of the scheme (M), as this enables them to feel like valued partners (O)
A Healthy Setting	36	When organisations adopt a settings-based approach in which university structures, policies, and processed are integrated with wider community health promotion (C), members will feel empowered to make healthy choices within a supportive environment (M), resulting in increased satisfaction & productivity for the whole university community (O)
	28	When organisations create a positive and enabling campus environment where mental health and wellbeing are supported (C), Students feel confident that they will not be stigmatized for accessing support (M) and engage readily with campus services (O)
	32	When organisations using a Healthy University approach (C), identifying the needs of their specific student populations (M), relevant programmes and interventions are developed (O)

## Results

### Document Characteristics

Overall, 57 documents were included in this Realist Review; none made explicit reference to social prescribing pathways operating within higher education. A range of mental health and wellbeing interventions were however identified; some of these functioned in isolation, whilst others were part of a broader initiative. Documents were from the United Kingdom (*n* = 18), United States (*n* = 17), Europe (*n* = 10), Australia (*n* = 6), Canada (*n* = 2), Asia (*n* = 2), Russia (*n* = 1) and Iran (*n* = 1).

### Main Findings

The focus of this review was to develop a programme theory explicating how social prescribing might be most optimally introduced to maximise benefit for both those using the services, and the organisations within which the pathways are hosted. Whilst literature relating directly to the use of social prescribing for HE students was limited, several studies articulated processes or pathways that were analogous to elements of the social prescribing process. Examples included using digital systems that linked those with wellbeing or mental health issues with services, mapping assets or resources within the university or wider community, and providing those undertaking a link worker role with effective training and support. Developing CMOCs from the literature also provided opportunities to examine social prescribing pathways created for other groups, e.g., older people living with chronic conditions, identifying demi-regularities [33, 34] that informed the programme theory developed within this project. Analysis of the data surfaced a programme theory in which four specific themes were supported by CMOCs, i.e., *An Accessible Gateway*, *A Skilled Peer*, *Trusted-Safe-Credible Resources*, and a *Healthy Setting.*

### An Accessible Gateway

When examining the literature surrounding systems or pathways for those experiencing mental health or wellbeing issues, the issue of routes into support begin to clearly surface as both an issue, and an area for careful consideration. The need for an accessible provision delivered both physically and virtually emerge as a means of reducing stigma associated with services, sharing resources, and (through a process of sharing experiences) developing positive coping strategies [11]. When services are delivered conventionally, students may find accessing these more difficult, particularly if they are not living on campus and only physically attend for limited periods during a week—issues that are further amplified in the current COVID-19 pandemic period. Web-based provision that includes an automated component may be useful in providing support for these groups [43, 44]. The range of provision is not always readily discernible when users are initially attempting to access the pathway, and this can be particularly problematic if services are offered by a range of departments within the university and beyond.

This issue was highlighted in a study examining the inception of social prescribing pathways within Primary Care, indicating that in order to increase engagement for all stakeholders, pathways should be integrated with existing systems as far as is reasonably practicable [11].

The range of mental health and wellbeing resources continues to increase [45], but those wishing to use the services may not be aware of the scope of provision, or appropriateness of pathways; a single platform in which these are located may be particularly useful in this respect [44, 46].

A number of documents in the review highlight the stigma associated with formally seeking support [24, 47–51], and this is increased for international students, or those from deprived socio-economic backgrounds [52]. These CMOCs indicate that digital or web-based resources may be particularly useful in ameliorating this issue, empowering students to access support without fear of negative perceptions or judgements.

Discussions around accessibility within the literature focus predominantly upon digital or web-based resources, but CMOCs within this theme also relate to the physical location, highlighting a number of considerations for any physical hubs [49]. Whilst there is a need to mirror digital provision in terms of centralising *information* regarding resources, the data also highlight the role that fellow students can play in raising awareness of wellbeing resources. Although physically locating student wellbeing services in one physical space may be convenient, the literature suggests that locating peers with knowledge of wellbeing resources within student-centric spaces such as cafés, libraries, or social learning spaces may increase fluency with the services that are available within and potentially beyond the institution [49].

### A Skilled Peer

The second theme emerging from this review focuses upon those facilitating the social prescription. The link worker or navigator role is complex, multifaceted, and continually evolving [15, 53], requiring a wide range of attributes including mental health first aid, safeguarding, advanced interpersonal skills and motivational interviewing, in addition to a comprehensive knowledge of the resources available locally [10, 54]. There is also significant variation in scope, ranging from the “signposting” of services through to a “holistic” model involving significant engagement with clients and intense support throughout the pathway [12]. The CMOCs developed within this theme reflect these challenges—highlighting the knowledge of the navigator, and the importance of trust and confidence in building a supportive relationship between user and link worker [11, 55]. Such attributes are not only integral in building relationships with users of the social prescribing pathway. In order to achieve “Buy In” from those who receive the referral, the navigator must also be viewed as skilled, competent, and capable of generating appropriate referrals to services [11].

When developing a social prescribing pathway within this setting, the value of peer involvement also emerges strongly; the sense of connection and sharing of a common experience translating into the normalising of mental health and wellbeing issues, and development of resilience [52]. The literature also surfaces the potentially stigmatising effect of engagement with conventional mental health services, indicating that a less ‘formal’ pathway with peer involvement may ameliorate this issue.

Peer input into a social prescribing pathway is viewed as accessible & authentic, facilitating the externalisation of wellbeing issues, and affording a sharing of coping strategies with the pathway user [56, 57].

Whilst the potential efficacy of a peer-led service is supported by the data, the literature also recognises not only the need for effective training, but the potential psychological pressure associated with the navigator role—with processes such as clinical supervision highlighted as important in buffering against these [11]. The review indicates that those undertaking the navigator role must be able to facilitate trusting relationships with those using the services and possess a knowledge of the available interventions. They must also have the ability to select interventions that are appropriate for each user of the pathway; failing to do this effectively will result in a loss of “buy in” and reduced engagement not only from users of the service, but also those providing social prescribing assets within the wider community. The potential psychological burden for those undertaking the navigator role is acknowledged, and buffering systems must be in place. The review data advocates peer involvement within a pathway, but how this may be operationalised is less clear given the issues around training and support.

### Trusted-Safe-Credible Resources

The literature and associated CMOCs here illustrate not only the importance of developing a pathway that is accessible and facilitated by an individual with a specific set of attributes, but also the quality of resources and assets that a user may be linked with. A basic web search for wellbeing or mental health resources will generate a significant number of hits, and the nature, scope, and quality of these is particularly challenging for those seeking support [50, 58]. Therefore, a need for resources that have been in some way curated and legitimised emerges from the review. Having access to resources that are perceived as being trustworthy not only increases engagement with services, but also contributes to an individual’s sense of agency and self-efficacy [59]. Consonant with findings in earlier themes, peer involvement within this curation process also emerges as being important for both resource sharing and network building—which then contributes to an increase in the social capital of individuals [46]. Other CMOCs supporting this theme relate to co-productive activity involving both users of the service and those providing assets and resources beyond the university. Cultivating these wider support networks increases the social capital of pathway users, embeds the university within the broader community, and acknowledges and values the contributions of partners [11, 60, 61].

This broader relationship between CMOCs may be perceived within this theme. Co-productive working between the university and wider asset/resource providers not only brings those *using* services together, but also fosters closely working relationships between those *delivering* them—developing networks, building social capital, and fostering a common sense of value and ambition. Providing credible resources that are peer-collated and shared, further contributes to this network building whilst increasing agency and overall self-efficacy for those using the pathway.

### A Healthy Setting

The final theme emerging from the review focuses upon the macro level institutional climate, articulating how this may impact upon the implementation and ongoing operation of a social prescribing pathway. A number of CMOCs developed echo the “Settings-based Approach” to health promotion outlined within the *Ottawa Charter* [62], and the “Healthy University” movement that emerged from this [63, 64]. The literature recognises that universities are environments in which a wide range of health and wellbeing initiatives are delivered; the “Healthy University” movement recognises this and focuses upon connecting potentially disparate strategies and mapping and connecting a diverse range of stakeholders within and beyond an organisation to address health and wellbeing. It is also worthy of note that the “Healthy University” movement, and the “Whole University Approach” [65, 66] associated with them not only highlight the importance of connected services within the organisation, but also across the wider community in which the university is situated—a finding that is highly congruent with the fundamental ethos of social prescribing [67].

The challenges faced by universities in terms of engaging students with services has emerged repeatedly within this review, particularly the sense of stigma experienced by those who may be accessing services, and the data highlights the enabling impact of a *Healthy Setting* upon this [68]. One must of course recognise that although key universal principles may be inducted from the literature, the profile of the organisation and its constituent community must also be carefully considered [63]. Thus, this final theme indicates that a social prescribing pathway will function most effectively when situated within a wider organisational culture that is settings-based and actively champions mental health and wellbeing. Services must be co-ordinated (both within the organisation and across the wider community in which the university is situated), and interventions and approaches must be responsive to the needs of the specific student population.

### The Refined Programme Theory

**FIGURE 2:**
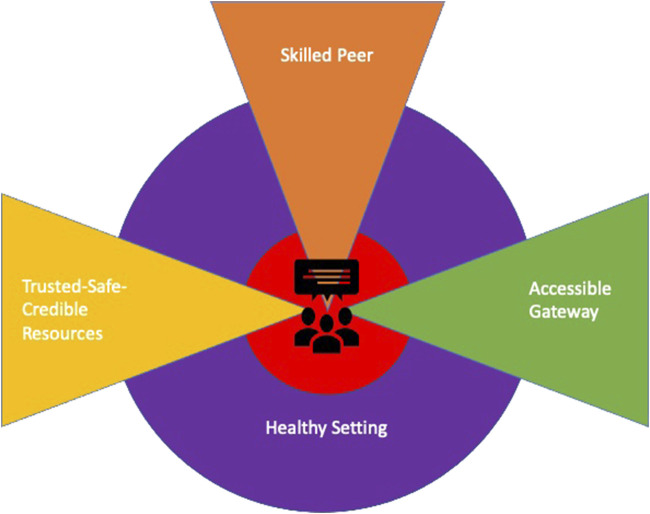
The refined programme theory (University of South Wales, United Kingdom. 2022).

The diagram presented within Figure 2 brings the themes discussed together into a Realist Programme Theory of social prescribing in HE. The student enters the system through an *Accessible Gateway* that may be physical or digital, and it is here that the individual providing the social prescription is initially encountered. The literature is replete with terms to classify both the scope and nature of this complex and multifaceted function [11, 54], but within this review the term “Navigator” is used. This *Skilled Peer* facilitates the social prescription through the “What Matters” conversation and/or signposts the student to a repository of *Trusted-Safe-Credible Resources*. These may include university services, curated information, assets located within the wider community, or a combination of these. Finally, the theory indicates that the social prescribing pathway sits within a wider *Healthy Setting* context that embodies a core ethos of accessibility, inclusivity, support, and empowerment for all members of the university community.

## Discussion

We sought to develop a Realist Programme Theory articulating the implementation of a social prescribing pathway within a HE environment. Whilst a range of isolated interventions that may be broadly captured as containing elements of a social prescription surfaced within the literature examined, e.g., an intergenerational arts project involving students and older adults from a local community [60], an online platform that allowed students to crowdsource wellbeing advice [46], we were unable to identify any bespoke pathways for university students. The review therefore represents a novel synthesis and application of the approach. Returning to the Refined Programme Theory (Figure 2), key findings may be drawn from the four themes within the review—relating to the pathway entry point, qualities of the social prescription “provider,” nature and scope of the resources that are available, and broader institutional context within which the pathway is situated. The key findings may be summarised.

### The Gateway

This is the first point of contact with the social prescribing pathway, and accessibility was a fundamental consideration. The review data indicate that there is a very strong preference for online access to wellbeing support which should be available at any time and from any location. As large and complex organisations, universities are comprised of many departments offering a range of services; the review acknowledges this, recommending that the social prescribing gateway be linked as seamlessly as possible with existing services—if a bespoke social prescribing IT solution is employed, this should be able to interface with existing systems. For the corresponding physical gateway, attention should be paid to the location. The literature highlights the stigma associated with accessing support for mental health and wellbeing [47, 49–51], and locating a physical hub within a “student owned” space such as a library or café will significantly ameliorate this issue.

### The Social Prescriber

The individual instigating a “What Matters” conversation and facilitating the subsequent social prescription is a pivotal component of the pathway. Several of the projects interrogated within the review involved mental health and wellbeing support provided by other students. However, the literature also recognises both the overall complexity and psychological challenges associated with the social prescriber role, and using volunteers in this way would be neither efficacious for the student or nascent social prescriber. Other ways in which peer support can be leveraged may therefore need to be considered when implementing social prescribing pathways, e.g., through peer-led wellness activities [57], or in some form of signposting or advisory capacity [12]. In considering the skills set of the social prescriber, the importance of a knowledgeable individual and well-developed interpersonal skills was evident [61]. However, one must also be cognisant that a social prescribing service requires the nurturing of community assets that exist beyond the university, and if “buy in” is to be achieved from these assets, then the prescriber will also need to cultivate significant Relational Social Capital [69–71], lest the pathway be no more than a signposting system [12] to services within the institution.

### The Resources

The third component of the model relates to the trusted and credible resources to which a user of the pathway is referred. The resources made available to the student may consist of services within the university (e.g., finance, counselling, student-run groups), local community assets (e.g., theatre groups, craft groups), digital resources (e.g., apps, online wellness platforms), or any combination of these. Asset mapping is a key process here; the social prescriber identifies potential support systems from within or beyond the university. The online space is also replete with support systems of variable quality, and the review indicates that attempting to discern what may be of genuine value can also be problematic when one is seeking support. For this reason, the review indicates curation of online resources to which those with lower levels of need may be signposted. Providing students with an opportunity to source, curate, and rate both physical and online assets can increase the sense of trust and authenticity for those using the services [46].

### The Wider Institutional Setting

The social prescribing pathway here sits within a wider institutional setting. The review data indicates that for a pathway to become more than a “pet” project or series of disparate interventions, the overarching institution must embody a clear commitment to physical and psychological wellbeing, as well as recognising its position as part of the wider community. Adopting such an approach may be challenging for some HEIs, but there is now a ground shift where the importance of a settings-based approach to physical and psychological wellbeing is recognised [63, 67,68].

### Revisiting the Review Questions

#### What Forms of Social Prescribing Interventions are Specifically Targeted at HE Students?

No specific social prescribing pathways directly targeting HE students were surfaced within this Realist Review. Several isolated interventions that contained elements of a social prescription were however identified, and these were drawn upon in terms of developing CMO configurations. The quality of any resources made available to students through social prescribing (*Trusted-Safe-Credible Resources*) features strongly within the review and has become an integral component of the Programme Theory.

#### How do HE Students Access Social Prescribing Interventions Targeting Them?

The need for an *Accessible Gateway* that spans the physical and virtual environments is pivotal here. Likewise, if one is to effectively gain access to social prescribing and its associated assets, the individual functioning in a social prescriber capacity must be a *Skilled Facilitator* who adopts a holistic approach; cultivating a meaningful relationship through a “what matters” conversation to co-produce a student-centred solution.

#### When do HE Students Access Social Prescribing Interventions?

The review data indicated that flexibility is paramount; this being particularly well served by the provision of both physical and virtual gateways.

#### For whom do Social Prescribing Interventions Work?

The interventions that were drawn upon to build the CMOCs and associated Programme Theory spanned a broad range of groups. It is therefore not possible at this stage to discern with any confidence for whom social prescribing interventions work.

#### To what Extent Does Social Prescribing Work for HE Students?

The extent to which social prescribing works for UK HE students was not discernible from the review data. The Realist Programme Theory here was subsequently tested with participants, and the findings will be reported within a future publication.

### Strengths and Limitations

This Rapid Realist Review explores the application of social prescribing within HE. This is a nascent area, and the data indicate the principles of social prescribing may impact positively upon student wellbeing, whilst also facilitating closer working relationships between universities and the wider communities within which they are situated. However, given the limited use of social prescribing approaches within HE, the range of literature and associated CMOCs was sparse, and there was a requirement to draw significant upon demi-regularities [33, 34] i.e., analogous processes functioning within other student wellbeing interventions. Whilst the direct generalisability of findings is confined to the UK Higher Education environment, it is anticipated that the underpinning principles can support the development of practice within the global arena.

### Conclusion

Contemporary literature suggests that supporting student with psychological wellbeing is an ongoing challenge; the review undertaken here indicates that leveraging the principles of social prescribing may be one strategy that could be used to ameliorate this. The review has resulted in the induction of a Realist Programme Theory of *causation* and *implementation*; articulating a pathway that functions specifically within the UK HE content. The underpinning principles may however be useful in the development of cognate approaches at a global scale.
